# Osteoprotegerin and MTHFR gene variations in rheumatoid arthritis: association with disease susceptibility and markers of subclinical atherosclerosis

**DOI:** 10.1038/s41598-022-13265-3

**Published:** 2022-06-09

**Authors:** Aikaterini Arida, Adrianos Nezos, Ioanna Papadaki, Petros P. Sfikakis, Clio P. Mavragani

**Affiliations:** 1 First Department of Propaedeutic Internal Medicine, National and Kapodistrian University of Athens Medical School, Athens, Greece; 2 Department of Physiology, School of Medicine, National and Kapodistrian University of Athens Medical School, Athens, Greece; 3 Department of Rheumatology, General Hospital of Athens “G. Gennimatas”, Athens, Greece; 4 Joint Academic Rheumatology Program, National and Kapodistrian University of Athens Medical School, Athens, Greece; 5 Rheumatology and Clinical Immunology Unit, Fourth Department of Internal Medicine, School of Medicine, University Hospital Attikon, NKUA, 12462 Haidari, Greece

**Keywords:** Cardiology, Molecular medicine, Rheumatology, Risk factors

## Abstract

We aimed to explore whether the rs2073618 variant (G1181C) of the osteoprotegerin (OPG) gene and the methylenetetrahydrofolate reductase (MTHFR) rs1801131 (A1298AC) and rs1801133 (C677T) gene polymorphisms contribute to rheumatoid arthritis (RA) susceptibility and RA related subclinical atherosclerosis. Overall 283 RA patients and 595 healthy controls (HC) were genotyped for common variants of the OPG and MTHFR genes using PCR based assays. Clinical and laboratory parameters were recorded following thorough chart review. Surrogate markers of subclinical atherosclerosis (Carotid/Femoral intima media thickness/plaque formation) along with traditional risk factors for atherosclerosis were assessed in all RA patients and 280HC. Increased prevalence of the CC genotype of the rs2073618 variant was detected in RA patients vs HC (42.4% vs. 33%, p-value: 0.04). RA patients with high serum titers of rheumatoid factor (RF) or anti-cyclic citrullinated peptide (CCP) antibodies displayed increased prevalence of the CC genotype of the rs2073618 variant of the OPG gene compared to HC (48.6% and 47.5 vs 33.3%, p-values: 0.0029and 0.0077 respectively). Of interest, this genotype turned to be associated with higher carotid IMT scores (0.872 ± 0.264 vs 0.816 ± 0.284, p-value: 0.01) and marginally with higher rates of carotid plaque formation (66% vs 54.1%, p = 0.06). The MTHFR 1298CC genotype was more prevalent only in the anti-CCP positive group compared to HC, with no associations detected with markers of subclinical atherosclerosis, following adjustment for traditional cardiovascular (CVD) risk factors. Reduced rates of carotid/femoral plaque formation were detected among RA patients harboring the MTHFR TT genotype (52.4 vs 72.7, p-value: 0.009, respectively). This association remained significant following adjustment for classical CVD risk factors (OR [95% CI 0.364 [0.173–0.765], p-value: 0.008). Genetic variations of the osteoprotegerin and MTHFR genes seem to increase susceptibility for seropositive RA and potentially contribute to subclinical atherosclerosis linked to RA. Larger studies are needed to confirm these findings.

## Introduction

Rheumatoid arthritis (RA) is a chronic systemic autoimmune disease, characterized by arthritis mainly affecting the peripheral joints, with considerable prevalence worldwide. It is well accepted that RA patients have increased morbidity and mortality due to accelerated cardiovascular disease (CVD) with respect to the general population, but the increased prevalence of traditional risk factors seem inadequate to fully account for the phenomenon^1–3^. Thus in recent years, great interest has been focused on the possible causal association between RA and CVD and the role of novel risk factors. Indeed, there is significant similarity between chronic inflammatory processes and the dysregulated immune response in RA and atheromatosis^2^. Moreover, recent studies have investigated the potential role of a few single nucleotide polymorphisms (SNPs) in various genes that are associated with CVD in the general population-such as methylenetetrahydrofolate reductase (MTHFR), TNFα, and ZC3HC1, NFKB or other SNPs such as rs964184 on atherosclerosis in RA^4^.

Osteoprotegerin (OPG) is a cytokine, member of the TNFR superfamily, that plays a role in the regulation of bone resorption^5,6^ as well as the development and progression of atherosclerosis, as OPG is actively involved in the development of vascular calcification^7^. We know from animal models that OPG-deficient mice develop, apart from early osteoporosis, arterial calcification and that OPG inactivation accelerates atherosclerotic lesion progression and calcification in ApoE−/− mice^8,9^. Along this side, numerous studies have designated that OPG represents an independent risk factor for progressive atherosclerosis and CVD, as OPG levels associate with increased carotid intima-media thickness (IMT) progression and coronary artery disease^10–12^.

Several polymorphisms of the gene encoding OPG (TNFRSF11B) mainly at promoter sites, have been associated with atherosclerosis in different patient populations; however, results from different studies are diverse^13–15^. While the RANKL/RANK/OPG system may promote RA inflammation and bone erosion, studies exploring the role of OPG genetic variants in patients with RA have not shown so far to increase disease susceptibility^16^. However, the CC genotype of the rs2073618, which encodes an Asn3Lys missense change in the osteoprotegerin gene (TNFRSF11B), maybe associated with coronary artery atherosclerosis^17^, while the GG genotype may be linked to reduced risk to develop cerebrovascular events in anti-cyclic citrullinated peptide (CCP) negative patients^18^.

It is well known that elevated homocysteine levels represent an independent risk factor for CVD in the general population^19–21^. The putative role of hyperhomocysteinemia (HHcy) in the development of atherosclerosis in autoimmune disorders has also been a field of interest, as it could partly explain the increased CVD risk in these patients^22–24^. Genetic polymorphisms of the MTHFR enzyme are a main cause of HHcy and have been associated with CVD in the general population^25–27^. Regarding RA, data is very limited and controversial, however there could be a potential role of MTHFR polymorphisms in the acceleration of atherosclerosis^4,28,29^.

In the present study, we aimed to explore whether the rs2073618 variant of the osteoprotegerin gene as well as the A1298C (rs1801131) and C677T (rs1801133) variant of the MTHFR gene contribute to RA related subclinical atherosclerosis.

## Results

### Prevalence of osteoprotegerin (OPG) and MTHFR gene variants in RA patients vs controls

Suppl. Tables S1 and S2 summarize the demographics, disease related characteristics, clinical features and indices in subclinical atherosclerosis in study participants. As shown in Table 1, the CC genotype increased disease susceptibility (adjustment for age and gender in the dominant model CC vs CG/GG OR [95% CI] 1.54 [1.03–2.27], p-value: 0.036). Further analysis revealed CG genotype of the rs2073618 OPG variant as a protective contributor against RA development in codominant and overdominant models (adjustment for age and gender ORs [95% CI] 0.54 [0.35–0.84], p-value: 0.017 and 0.57 [0.38–0.84], p-value: 0.0048, respectively). While no statistically significant differences between the groups were detected for the MTHFR C677T, the CC genotype of the MTHFR A1298Cvariant was more frequent among RA patients (adjustment for age and gender in the recessive model genotype CC vsAA/AC: OR [95% CI] 1.77 [1.05–2.98], p-value: 0.031).

**Table 1:** Prevalence of the OPG rs2073618 and the MTHFR rs1801133 and rs1801131 SNP genotypes in RA patients and HC, adjusted by gender and age.

SNPs	Genotype	HAPMAP (European) Database (%)	HC, n (%)	RA, n (%)	ORcodominant model [95%CI]	p-value	ORdominant model [95%CI]	p-value	ORrecessive model [95%CI]	p-value	ORover dominant model [95%CI]	p-value	OR log additive model [95%CI]	p-value
OPG rs2073618					**CC VS CG VS GG**		**CC VS (CG/GG)**		**(CC/CG) VS GG**		**CG VS (CC/GG)**			
	**CC**	29	78 (33.3)	111 (42.7)	1.00	**0.017**	**1.54 (1.03–2.27)**	**0.036**	1.22 (0.77–1.93)	0.4	**0.57 (0.38–0.84)**	**0.0048**	0.89 (0.70–1.15)	0.38
	**CG**	53	107 (45.7)	85 (32.7)	**0.54 (0.35–0.84)**									
	**GG**	18	49 (20.9)	64 (24.6)	0.89 (0.53–1.49)									
MTHFR rs1801133 (C677T)					**CC VS CT VS TT**		**CC VS (CT/TT)**		**(CC/CT) VS TT**		**CT VS (CC/TT)**			
	**CC**	46.9	141 (34.6)	106 (37.9)	1.00	0.47	0.82 (0.59–1.13)	0.23	0.87 (0.57–1.33)	0.52	0.90 (0.66–1.22)	0.49	0.87 (0.70–1.09)	0.24
	**CT**	44.2	200 (49.1)	131 (46.8)	0.83 (0.59–1.17)									
	**TT**	8.8	66 (16.2)	43 (15.8)	0.78 (0.49–1.25)									
MTHFR rs1801131 (A1298C)					**AA VS AC VS CC**		**AA VS (AC/CC)**		**(AA/AC) VS CC**		**AC VS (AA/CC)**			
	**AA**	43.4	198 (48.6)	128 (45.7)	1.00	0.096	1.14 (0.83–1.55)	0.41	**1.77 (1.05–2.98)**	**0.031**	0.93 (0.68–1.27)	0.64	1.21 (0.96–1.54)	0.11
	**AC**	45.1	176 (43.2)	118 (42.1)	1.03 (0.74–1.43)									
	**CC**	11.5	33 (8.1)	34 (12.1)	**1.79 (1.04–3.09)**									

Genotypes, OR and p-value for the five genetic models (codominant, dominant, recessive, overdominant and additive) were estimated with SNPstats software (statistically significant if p < 0.05).

*OPG* osteoprotegerin, *MTHFR* methylene tetrahydrofolate reductase, *OR* odds ratio, *SNP* single nucleotide polymorphism, *VS* versus, *RA* rheumatoid arthritis, *HC* healthy controls, *HAPMAP* haplotype map.

Significant values are in bold.

### Associations of OPG and MTHFR variants with disease related features and markers of subclinical atherosclerosis among RA patients

We next wished to explore whether OPG and MTHFR variants are associated with distinct clinical and serological characteristics, as well as with markers of subclinical atherosclerosis among RA patients. Thus, compared to RA patients sharing the CG/GG genotype, those with the CC genotype of the rs2073618 OPG variant were found to display increased rates of RF positivity and higher IMT values (69.9 vs 55.1, p = 0.02 and 0.872 ± 0.264 vs 0.816 ± 0.284, p = 0.01, respectively). A trend towards higher DAS28 levels and rates of carotid atherosclerotic plaque formation was also observed among RA patients with the CC vs the CG/GG genotypes (3.5 ± 1.4 vs 3.2 ± 1.3 and 66% vs 54.1%, p-values: 0.06, for both comparisons). No other differences in disease related features or classical CVD risk factors between the CC and the GG/GC genotype group were detected (Table 2). Considering available variables included in the Expanded Cardiovascular Risk Prediction Score for Rheumatoid Arthritis ERS-RA score^30^ a multivariate model was constructed revealing diabetes, disease duration and DAS28 as independent predictors for arterial wall thickening [ORs 95% CI 1.037 (1.008–1.067), 1.789 (1.413–2.266) and 2.078 (0.953–4.532), respectively].

**Table 2:** Associations of osteoprotegerin (OPG) and methylene-tetrahydrofolate reductase (MTHFR) minor allele homozygous genotypes with demographics, disease related features, traditional CV risk factors and markers of subclinical atherosclerosis among rheumatoid arthritis (RA) patients.

	*OPG rs2073618*			*MTHFR rs1801133 (C677T)*			*MTHFR rs1801131 (A1298C)*		
	CC (n = 111)	CG/GG (n = 151)	p-value	TT (n = 43)	TC/CC (n = 239)	p-value	CC (n = 35)	AC/AA (n = 247)	p-value
Age (Mean ± SD)	60.4 ± 12	61.1 ± 11.6	ns	61.6 ± 10.5	62 ± 12.2	ns	59.3 ± 11	62.3 ± 12	ns
Gender (male)(%)	12.6	18.5	ns	16.3	14.6	ns	85.7	85	ns
BMI (kgs/m^2^)	28.5 ± 5.8	27.8 ± 5.1	ns	30.2 ± 6.4	27.8 ± 5.2	**0.02**	27.3 ± 4.5	28.2 ± 5.6	ns
Disease duration	14.2 ± 10.2	13.2 ± 10.0	ns	13.4 ± 9.6	13.8 ± 10.3	ns	16 ± 12.6	13.4 ± 9.8	ns
RF positivity (%)	69.9	55.1	**0.02**	57.9	62.8	ns	61.3	62.2	ns
Anti-CCP positivity (%)	65.1	54.4	ns	63.2	58.1	ns	63.3	58.3	ns
ESR (mean ± SD)	26.5 ± 23.3	24.1 ± 19.7	ns	21.2 ± 18.7	27 ± 22.4	ns	24.5 ± 16.6	26.3 ± 22.6	ns
DAS 28 (mean ± SD)	3.5 ± 1.4	3.2 ± 1.3	0.06	3.0 ± 1.2	3.5 ± 1.5	ns	3.4 ± 1.1	3.5 ± 1.5	ns
Diabetes (%)	9.9	4	ns	4.7	7.5	ns	5.7	7.3	ns
Hypertension (%)	46.9	42	ns	44.2	43.5	ns	40	44.1	ns
Current Smokers (%)	21.6	30.7	ns	9.3	29.7	0.005	34.3	25.5	ns
Total Cholesterol (mg/dl)	201.1 ± 35.6	204.5 ± 37.8	ns	204.3 ± 33.8	202.4 ± 36.8	ns	212.4 ± 39.5	201.5 ± 35.8	ns
LDL (mg/dl)	119.9 ± 30.6	120.9 ± 30.7	ns	120.3 ± 27.2	120.4 ± 31.1	ns	127.7 ± 35	119.4 ± 29.8	ns
Carotid IMT (mean ± SD)	0.872 ± 0.264	0.816 ± 0.284	**0.01**	0.803 ± 0.294	0.872 ± 0.333	ns	0.780 ± 0.270	0.874 ± 0.334	0.049
Plaques									
Carotid and/or femoral	72	65.5	ns	52.4	72.7	0.009	60	70.9	ns
Carotid	66	54.1	0.06	47.6	63.4	0.05	48.6	62.8	ns
Femoral	59	51.4	ns	42.9	57.7	0.08	48.6	56.4	ns

Following stratification of RA patients according to RF status and adjustment for age and sex, a significantly increased frequency of the CC genotype was detected in the RF positive RA group compared to both HC control group (Table 3) and RF negative RA subset (Table 4) [48.6% vs 33.3%, OR [95% CI] 1.89 [1.25–2.86], p-value: 0.0029 and 2.0 [1.16–3.45], p-value: 0.012, respectively, in dominant models). Moreover, decreased frequency of the CG genotype in the RF positive RA group compared to both HC (Table 3) and RF negative patients (Table 4) [27.7% vs 45.7% vs 40.9%, p-values: 0.0004 and 0.032, respectively, in the overdominant model]. No significant differences were found between HC and RF negative groups (Suppl. Table S3).

**Table 3:** Prevalence of the OPG rs2073618 and the MTHFR rs1801133 and rs1801131 SNP genotypes in RA RF positive patients and HC, adjusted by gender and age.

SNPs	Genotype	HAPMAP (European) database (%)	HC, n (%)	RF positive RA, n (%)	OR codominant model [95%CI]	p-value	OR dominant model [95%CI]	p-value	OR recessive model [95%CI]	p-value	OR overdominant model [95%CI]	p-value	OR logadditive model [95%CI]	p-value
OPG rs2073618					**CC VS CG VS GG**		**(CG/GG) VS CC**		**(CC/CG) VS GG**		**CG VS (CC/GG)**			
	**CC**	29	78 (33.3)	72 (48.6)	1.00	**0.0011**	**1.89 (1.25–2.86)**	**0.0029**	1.17 (0.71–1.91)	0.53	**0.45 (0.29–0.71)**	**0.0004**	0.80 (0.61–1.06)	0.11
	**CG**	53	107 (45.7)	41 (27.7)	**0.42 (0.26–0.67)**									
	**GG**	18	49 (20.9)	35 (23.6)	0.77 (0.45–1.33)									
MTHFR rs1801133 (C677T)					**CC VS CT VS TT**		**CC VS (CT/TT)**		**(CC/CT) VS TT**		**CT VS (CC/TT)**			
	**CC**	46.9	141 (34.6)	56 (34.6)	1.00	0.5	0.93 (0.63–1.38)	0.71	0.73 (0.43–1.24)	0.24	1.10 (0.76–1.60)	0.61	0.88 (0.67–1.16)	0.38
	**CT**	44.2	200 (49.1)	84 (51.9)	1.00 (0.66–1.51)									
	**TT**	8.8	66 (16.2)	22 (13.6)	0.73 (0.41–1.31)									
MTHFR rs1801131 (A1298C)					**AA VS AC VS CC**		**AA VS (AC/CC)**		**(AA/AC) VS CC**		**AC VS (AA/CC)**			
	**AA**	43.4	198 (48.6)	75 (46.3)	1.00	0.18	1.11 (0.77–1.61)	0.58	1.81 (0.98–3.37)	0.064	0.91 (0.62–1.32)	0.61	1.20 (0.90–1.60)	0.21
	**AC**	45.1	176 (43.2)	68 (42)	1.00 (0.67–1.48)									
	**CC**	11.5	33 (8.1)	19 (11.7)	1.81 (0.95–3.46)									

Genotypes, OR and p-value for the five genetic models (codominant, dominant, recessive, overdominant and additive) were estimated with SNPstats software (statistically significant if p < 0.05).

*OPG* osteoprotegerin, *MTHFR* methylene tetrahydrofolate reductase, *OR* odds ratio, *SNP* single nucleotide polymorphism, *VS* versus, *RA* rheumatoid arthritis, *HC* healthy controls, *RF* rheumatoid factor, *HAPMAP* haplotype map.

Significant values are in bold.

**Table 4:** Prevalence of the OPG rs2073618 and the MTHFR rs1801133 and rs1801131 SNP genotypes in RA RF negative and RF positive patients, adjusted by gender and age.

SNPs	Genotype	HAPMAP (European) Database (%)	RA RF negative, n (%)	RA RF positive, (%)	OR codominant model [95%CI]	p-value	OR dominant model [95%CI]	p-value	OR recessive model [95%CI]	p-value	OR overdominant model [95%CI]	p-value	OR logadditive model [95%CI]	p-value
OPG rs2073618					**CC VS CG VS GG**		**CC VS (CG/GG)**		**(CC/CG) VS GG**		**CG VS (CC/GG)**			
	**CC**	29	31 (33.3)	72 (48.6)	1.00	**0.03**	**2.0 (1.16–3.45)**	**0.012**	0.85 (0.46–1.56)	0.6	**0.55 (0.31–0.95)**	**0.032**	0.73 (0.53–1.02)	0.066
	**CG**	53	38 (40.9)	41 (27.7)	**0.44 (0.24–0.83)**									
	**GG**	18	24 (25.8)	35 (23.6)	0.58 (0.30–1.15)									
MTHFR rs1801133					**CC VS CT VS TT**		**CC VS (CT/TT)**		**(CC/CT) VS TT**		**CT VS (CC/TT)**			
	**CC**	46.9	42 (42.4)	56 (34.6)	1.00	0.34	1.33 (0.79–2.24)	0.28	0.81 (0.40–1.64)	0.56	1.46 (0.88–2.44)	0.14	1.09 (0.75–1.58)	0.65
	**CT**	44.2	41 (41.4)	84 (51.9)	1.46 (0.84–2.55)									
	**TT**	8.8	16 (16.2)	22 (13.6)	1.00 (0.46–2.15)									
MTHFR rs1801131					**AA VS AC VS CC**		**AA VS (AC/CC)**		**(AA/AC) VS CC**		**AC VS (AA/CC)**			
	**AA**	43.4	44 (44.4)	75 (46.3)	1.00	0.92	0.91 (0.55–1.51)	0.72	1.02 (0.47–2.23)	0.96	0.90 (0.54–1.50)	0.69	0.96 (0.66–1.39)	0.81
	**AC**	45.1	43 (43.4)	68 (42.0)	0.89 (0.52–1.54)									
	**CC**	11.5	12 (12.1)	19 (11.7)	0.97 (0.43–2.21)									

Genotypes, OR and p-value for the five genetic models (codominant, dominant, recessive, overdominant and additive) were estimated with SNPstats software (statistically significant if p < 0.05).

*OPG* osteoprotegerin, *MTHFR* methylene tetrahydrofolate reductase, *OR* odds ratio, *SNP* single nucleotide polymorphism, *VS* versus, *RA* rheumatoid arthritis, *RF* rheumatoid factor, *HAPMAP* haplotype map.

Significant values are in bold.

After RA patients were stratified according to anti-CCP status and adjustment for age and sex, genotype analysis revealed a statistically increased prevalence of the CC genotype of rs2073618 OPG SNP in anti-CCP positive RA patients compared to HC (Table 5) (OR [95% CI] 1.89 [1.18–3.03], p-value: 0.0077, in the dominant model). Moreover, anti-CCP positive RA group displayed decreased frequency of the CG genotype compared to HC [32.6% vs 45.7%, p-value: 0.018, in the overdominant model] (Table 5), as well as of the GG genotype compared to anti-CCP negative counterparts (19.9 vs 31.6, p-value: 0.04 in the recessive model, Table 6). Decreased frequency of the CG genotype in anti-CCP negative RA patients compared to HC [31.6% vs 45.7%, p-value: 0.013, in the overdominant model] was also found (Suppl. Table S4).

**Table 5:** Prevalence of the OPG rs2073618 and the MTHFR rs1801133 and rs1801131 SNP genotypes in RA anti-CCP positive patients and HC, adjusted by gender and age.

SNPs	Genotype	HAPMAP (European) database (%)	HC, n (%)	RA anti-CCP positive, n (%)	OR codominant model [95% CI]	p-value	OR dominant model [95%CI]	p-value	OR recessive model [95% CI]	p-value	OR overdominant model [95% CI]	p-value	OR logadditive model [95% CI]	p-value
OPG rs2073618					**CC VS CG VS GG**		**CC VS (CG/GG)**		**(CC/CG) VS GG**		**CG VS (CC/GG)**			
	CC	29	78 (33.3)	67 (47.5)	1.00	**0.021**	**1.89 (1.18–3.03)**	**0.0077**	0.90 (0.51–1.58)	0.71	**0.57 (0.35–0.91)**	**0.018**	0.74 (0.54–1.01)	0.052
	CG	53	107 (45.7)	46 (32.6)	**0.49 (0.29–0.81)**									
	GG	18	49 (20.9)	28 (19.9)	0.63 (0.34–1.17)									
MTHFR rs1801133 (C677T)					**CC VS CT VS TT**		**CC VS (CT/TT)**		**(CC/CT) VS TT**		**CT VS (CC/TT)**			
	CC	46.9	141 (34.6)	59 (38.6)	1.00	0.57	0.81 (0.55–1.20)	0.29	0.89 (0.53–1.49)	0.65	0.88 (0.60–1.28)	0.50	0.87 (0.66–1.15)	0.34
	CT	44.2	200 (49.1)	70 (45.8)	0.82 (0.54–1.24)									
	TT	8.8	66 (16.2)	24 (15.7)	0.79 (0.45–1.10)									
MTHFR rs1801131 (A1298C)					**AA VS AC VS CC**		**AA VS (AC/CC)**		**(AA/AC) VS CC**		**AC VS (AA/CC)**			
	AA	43.4	198 (48.6)	72 (47.1)	1.00	0.14	1.07 (0.74–1.57)	0.71	**1.87 (1.01–3.47)**	**0.05**	0.86 (0.58–1.26)	0.43	1.19 (0.89–1.59)	0.24
	AC	45.1	176 (43.2)	62 (40.5)	0.95 (0.63–1.42)									
	CC	11.5	33 (8.1)	19 (12.4)	1.83 (0.96–3.48)									

Genotypes, OR and p-value for the five genetic models (codominant, dominant, recessive, overdominant and additive) were estimated with SNPstats software (statistically significant if p < 0.05).

*OPG* osteoprotegerin, *MTHFR* methylene tetrahydrofolate reductase, *OR* odds ratio, *SNP* single nucleotide polymorphism, *VS* versus, *RA* rheumatoid arthritis, *HC* healthy controls, *anti-CCP* anti-cyclic citrullinated peptide, *HAPMAP* haplotype map.

Significant values are in bold.

**Table 6:** Prevalence of the OPG rs2073618 and the MTHFR rs1801133 and rs1801131 SNP genotypes in RA anti-CCP negative and anti-CCP positive patients, adjusted by gender and age.

SNPs	Genotype	HAPMAP (European) Database (%)	RA anti-CCP negative, n (%)	RA anti-CCP positive, n (%)	OR codominant model [95% CI]	p-value	OR dominant model [95% CI]	p-value	OR recessive model [95% CI]	p-value	OR overdominant model [95% CI]	p-value	OR logadditive model [95% CI]	p-value
OPG rs2073618					**CC VS CG VS GG**		**CC VS (CG/GG)**		**GG VS (CC/CG)**		**CG VS (CC/GG)**			
	CC	29	36 (36.7)	67 (47.5)	1.00	0.093	0.64 (0.38–1.09)	0.10	**0.54 (0.30–0.97)**	**0.04**	1.05 (0.60–1.82)	0.86	**0.70 (0.51–0.97)**	**0.034**
	CG	53	31 (31.6)	46 (32.6)	0.80 (0.43–1.47)									
	GG	18	31 (31.6)	28 (19.9)	**0.49 (0.25–0.93)**									
MTHFR rs1801133 (C677T)					**CC VS CT VS TT**		**CC VS (CT/TT)**		**(CC/CT) VS TT**		**CT VS (CC/TT)**			
	CC	46.9	38 (35.5)	59 (38.6)	1.00	0.63	0.87 (0.52–1.45)	0.59	1.24 (0.61–2.52)	0.55	0.79 (0.48–1.30)	0.35	0.99 (0.69–1.42)	0.94
	CT	44.2	55 (51.4)	70 (45.8)	0.81 (0.47–1.39)									
	TT	8.8	14 (13.1)	24 (15.7)	1.10 (0.51–2.39)									
MTHFR rs1801131 (A1298C)					**AA VS AC VS CC**		**AA VS (AC/CC)**		**(AA/AC) VS CC**		**AC VS (AA/CC)**			
	AA	43.4	47 (43.9)	72 (47.1)	1.00	0.65	0.88 (0.53–1.44)	0.60	1.25 (0.57–2.75)	0.58	0.80 (0.48–1.32)	0.38	0.98 (0.68–1.41)	0.9
	AC	45.1	49 (45.8)	62 (40.5)	0.82 (0.48–1.39)									
	CC	11.5	11 (10.3)	19 (12.4)	1.13 (0.49–2.60)									

Genotypes, OR and p-value for the five genetic models (codominant, dominant, recessive, overdominant and additive) were estimated with SNPstats software (statistically significant if p < 0.05).

*OPG* osteoprotegerin, *MTHFR* methylene tetrahydrofolate reductase, *OR* odds ratio, *SNP* single nucleotide polymorphism, *VS* versus, *RA* rheumatoid arthritis, *anti-CCP* anti-cyclic citrullinated peptide, *HAPMAP* haplotype map.

Significant values are in bold.

As for the MTHFR polymorphisms, anti-CCP positive, but not anti-CCP negative RA patients following adjustment for age and gender, depicted increased frequency of the MTHFR 1298 CC genotype compared to HC (OR [95% CI]: 1.87 [1.01–3.47], p-value: 0.05, recessive model, Table 5 and Suppl. Table S4). No differences in MTHFR variants between anti-CCP positive and negative RA groups were detected (Table 6). Moreover, no significant associations between MTHFRC677T variants with disease susceptibility or related features were detected (Tables 1 and 2).

Of interest, RA individuals carrying the TT genotype of the C677T variant were less likely to smoke (9.3 vs 29.7, p-value: 0.005), had higher BMI levels (30.2 ± 6.4 vs 27.8 ± 5.2, p-value: 0.02) and reduced rates of carotid/femoral plaque formation (52.4 vs 72.7, p-value: 0.009, respectively, Table 2). This association remained significant following adjustment for classical CVD risk factors (OR [95% CI 0.364 [0.173–0.765], p-value: 0.008). Moreover, the A1298CCC genotype was found to be associated with reduced carotid IMT values (0.780 ± 0.270 vs 0.874 ± 0.334 mm, p-value: 0.049). The latter associations were not retained following adjustment for classical CVD contributors (data not shown).

The prevalence of OPG and MTHFR variants between RF and or anti-CCP positive patients compared to HC and their negative counterparts was calculated. Supplementary Tables S5 and S6 depict the comparisons between RF and or anti-CCP positive patients versus HC and their negative counterparts, respectively. No statistically significant associations were detected in all models implemented.

## Discussion

In the present study we focused on the possible implication of genotypes of the osteoprotegerin gene and the MTHFR gene on the acceleration of atherosclerosis in RA. We found that seropositive RA patients (either for RF or anti-CCP) display increased prevalence of the CC genotype of the rs2073618 variant of the OPG gene compared to HC. Of interest, this genotype turned to be associated with higher carotid IMT scores and marginally with carotid plaque formation. Moreover, the MTHFR 1298CC genotype was more prevalent only in the anti-CCP positive group compared to HC and negatively associated with indices of subclinical CVD. These associations were not retained following adjustment for traditional CVD predictors. In contrast, a negative association between MTHFR TT and plaque formation was detected, following adjustment for CVD risk factors.

It is well established that the RANKL/RANK/OPG system plays a role in RA disease pathogenesis and that RA patients have significantly higher circulating OPG levels compared to controls in association with disease activity^31–33^. While our results designated a protective role of the CG genotype of the rs2073618 OPG variant against RA development, two recent meta-analysis did not relate variants of the OPG genes to RA susceptibility, whereas the rs2073618 variant was associated with erosions in a French RA cohort^16,34,35^. Moreover, increased OPG levels cause artery calcification and atherosclerosis and thus associate with CVD in the general population and in RA patients^10,12,36,37^. However, only specific variants of the OPG gene have been associated with atherosclerosis and CVD in non-rheumatic patients in few studies^38–40^. In RA, only two studies have examined the association of different variants of the OPG gene and atherosclerosis and results are somewhat inconsistent. In accordance with our results, Chung et al. found that the CC genotype of the rs2073618 was associated with coronary artery calcium, whereas another study failed to associate any of the examined variants with the risk of CV events and designated a possible protective effect of the OPG CGA haplotype on CV risk in the anti-CCP negative RA subset^17,18^.

The association between the MTHFR gene variants -mainly C677T but also A1298C-and CVD has been widely investigated in the general population and it is thought that individuals with the *MTHFR*677TT genotype had a significantly higher risk of CVD^26,41–43^. This association has also been a field of interest for patients with autoimmune disorders, especially systemic lupus erythematosus, where the MTHFR677TT genotype emerged as an independent predictor for both plaque formation and arterial wall thickening^44^. In the setting of RA, HHcy could partially account for the heightened CVD risk, as there are higher Hcy concentrations in RA patients with a history of CVD compared to controls or RA patients without CVD in association with atherosclerosis in RA^24,45^. However, since methotrexate treatment and folic acid supplementation could alter Hcy levels, the contribution of the MTHFR gene polymorphisms in HHcy and the development of CVD is yet unclear^46^. In two studies, RA patients with the 677TT genotype had higher plasma homocysteine levels than those with 677CC genotype, and this was also associated with increased cIMT^28,47^. Moreover, TT genotype and T allele were also linked to susceptibility to develop RA, probably indicating a genetic interaction between RA and CVD^28^. However, another study failed to show an association between the C677T variant and CV events or subclinical atherosclerosis, whereas the MTHFR 1298 allele C frequency was increased in patients with CV events, and patients carrying the MTHFR 1298 AC and CC genotypes had a significantly decreased flow-mediated endothelium-dependent vasodilatation than those with the 1298AA genotype^48^. In contrast, Davis et al. supported there is no association of MTHFR polymorphisms with CV events in RA, and that CV events are associated with traditional risk factors and methotrexate use^29^. Interestingly, a recent study showed that the expression of the MTHFR gene is down-regulated in patients with RA compared to controls, especially those with ischemic heart disease, which could suggest a potential implication of the transcriptional regulation of MTHFR in the pathogenesis of RA^49^.

While we focused on only these two specific genes and their association with CVD in RA, many other single nucleotide polymorphisms (SNPs)—that have been associated with CVD in the general population- have been investigated in recent years for their possible impact on the progression of atherosclerosis in RA. A recent review by López-Mejías et al. summarized all knowledge on the genetic influence in the risk of subclinical atherosclerosis and CV disease in RA through 2016, where specific SNPs were associated with acceleration of atherosclerosis in RA^4^. Since then, few other studies have enriched our knowledge on the contribution of variants of specific genes, apart from MTHFR and OPG, in acceleration of CVD in RA.

In RA, the low activity of paraoxonase 1 (PON1) is related to a more atherogenic lipid in these patients and the implication of some polymorphisms of the PON1 gene has been described recently with apparently contradictory results^4,50^. A recent study examining three polymorphisms of the PON1 genes (−108C>T, L55M, and Q192R), associated the haplotype TLQ with reduced PON1 activity and PON1 levels, and thus with a more atherogenic lipid profile in RA^51^. In accordance with previous studies that have linked QQ genotype with reduced PON activity and higher prevalence of CV events, a Spanish study confirmed that the Q192R is involved in reduced enzymic activity, however that does not apply for the L155M^52–54^. Moreover, Atwa et al. showed that the QQ genotyping was associated with increased cIMT & plaques, while another study linked the RR genotype with reduced CV risk, as previously supported for carotid plaques^55–57^.

We retrieved three studies examining the link between apolipoprotein E (ApoE) polymorphisms, a lipoprotein that plays a role in lipid metabolism^58^ and CVD in RA. In all three, ApoE genotypes distribution was similar between RA patients and HC and all reported an association between the ε4 allele to a worse, more atherogenic lipid profile. However, no ApoE genotype was associated with CVD or the presence of plaques or carotid IMT in previous studies^59,60^, whereas a recent study by Chen et al. claimed that patients with the ε3ε4 genotype presented with higher CVD risk^61^.

Nitric oxide synthases (NOS) are enzymes catalyzing the production of NO and some NOS genes polymorphisms, by reducing NOS expression and endothelial NO synthesis and availability in the vessel, have been associated with endothelial dysfunction and pathogenesis of atherosclerosis^62,63^. In addition, some variants of the NOS genes have been associated with RA susceptibility^64–66^, however it is not clear whether these polymorphisms have a direct impact on CVD risk in RA^67^. Gonzalez-Gay et al. designated a possible interaction between HLA-DRB1*0404 allele and the NOS3 (-786) TT genotype, that could increase the risk of CV events in RA patients^68^. On the other hand, a recent study by Luo et al. showed that patients with the CC genotype had lower flow-mediated dilation and that the down-expression of -786T>C is associated with an increased risk of endothelial dysfunction in RA^69^. Finally, Dimitroulas et al., in the same concept of reduced NO plasma levels and consequent impaired vascular homeostasis, investigated the effect of Alanine-glyoxylate aminotransferase 2 (AGTX2) gene polymorphisms on the levels Asymmetric (ADMA) and symmetric (SDMA) dimethylarginines in patients with RA, but found no association between serum concentrations of dimethylarginines and genetic variants of the AGXT2 gene^4,70,71^.

Numerous studies have also focused on the potential impact of genes related to specific cytokines on the progression of CVD in RA^4^. A recent study associated an IL-19 risk allele, rs17581834 (T), with stroke/myocardial infarction in patients with SLE and RA, but not HC, implying a shared immune pathway in the pathogenesis of immune diseases and CVD^72^. Another study examining the role of IL-32 genes, found that, while the distribution of alleles was similar between RA patients and controls, subjects homozygous for the C allele had higher levels of high density lipoprotein cholesterol (HDLc), suggesting a protective role against CVD. On contrary, the CC-genotype was associated with elevated low density lipoprotein cholesterol (LDLc) and total cholesterol (TC) in individuals with plaques^73^. Farias et al. found no association between two polymorphisms (-607C/A and -137G/C) of the IL-18 gene and RA nor risk factors for CVD; similarly no influence was found of the rs1058587 SNP within GDF15 (MIC1) gene on the development of CVD^74,75^. Although discrepant with a previous report^76^, Agca et al. examined the effects of the Interferon regulatory factor 5 (IRF5) gene polymorphisms (rs2004640 and rs4728142) and found that some genotypes were associated with cIMT and cIMT progression, but not CV events in RA patients, implicating a role of the IRF5 transcription pathway in atherosclerosis^77^.

Given the association of CRP levels and CVD in the general population, as well as in patients with RA, some studies evaluated the impact of CPR gene polymorphisms on CV risk^78–84^. Regarding RA, Ibrahim et al. examined three SNPs (rs1205, rs1800947 and rs3091244) and found no association between any of the haplotypes investigated and all cause or CVD mortality^85^. Similarly, a recent study by Lopez-Merijas et al. found no association between evaluated genes that influence CRP levels, and the presence of CV events, carotid plaques or cIMT^86^. In contrast, another study evaluating the genetic variants NLRP3-Q705K and CARD8-C10X related to the NLRP3 inflammasome, showed that the NLRP3-Q705K minor allele was associated with an increased risk of stroke/transient ischemic attack, but not myocardial infarction (MI)/angina pectoris, while CARD8-C10X was not associated with any type of CV event^87^.

Regarding other genes, a recent genome-wide association study on almost 3000 RA patients suggested that the minor allele G of the rs116199914 variant in the RARB (Retinoic Acid Receptor Beta) gene is associated with cIMT and possibly contributes to the development of subclinical atherosclerosis in patients with RA^88^. Another large study evaluating 12 SNPs in RA patients, identified novel associations between subclinical atherosclerosis and variants in SLC17A2 (rs17526722), PPCDC (rs1867148), COL4A1 (rs496916) and SLCA13 (rs515291) genes, that may constitute new candidate risk loci for CVD in RA^89^. Evaluation of variants of the platelet endothelial cell adhesion molecule-1 (PECAM-1) gene showed that, there was no association between genotype and atherosclerotic complications, as opposed to patients with SLE^90^. Finally, two other studies failed to designate any role of the Asp299Gly Toll-like receptor 4 polymorphism on endothelial dysfunction, or an association of the rs10116277 or rs1537375 SNPs of the 9p21.3 genomic region with cIMT or carotid plaques^91,92^.

Interestingly, few studies, while not directly evaluating CVD in RA, associated polymorphisms of specific genes with increased prevalence of classical CVD risk factors. Regarding dyslipidemia, Davis et al. examined SNPs associated with RA susceptibility with lipid levels in RA and found that the REL SNP rs9309331 homozygous minor allele was associated with higher LDL levels^93^. On the antipode, alleles of SNPs modulating low-density lipoprotein (LDL) cholesterol were associated with disease risk, activity and severity, thus implying the presence of common genetic mechanisms^94^. In respect to blood pressure (BP) levels and the prevalence of hypertension, Panoulas et al. showed that RA patients who were CC homozygotes for the galectin-2 (LGALS2) 3279 C/T single nucleotide polymorphism (SNP) had higher diastolic BP that the TT homozygotes. The same researchers found that the T allele of the TGFB1 869T/C and the rs1800541-rs5370 T-T EDN1 haplotype were associated with increased risk of hypertension^95–97^. Finally, given the effect of MTX therapy on BP levels, a recent study investigated the association between the ATP-binding cassette efflux transporter gene *ABCG2* (rs2231142) SNP and BP and arterial stiffness in RA and found that rs2231142 heterozygotes (AC) had significantly lower age-adjusted clinical systolic BP levels when compared to the CC group, however there was no difference in Augmentation Index (AIx) or Pulse Wave Velocity (PWV)^98^.

Our study has some limitations. Firstly, it is not a single-center study and ultrasounds evaluating subclinical atherosclerosis were not performed by one technician. Secondly, we did not measure homocysteine and OPG levels in our patients, however, we believe that the association of the SNPs under study and the levels of Hcy and OPG are well described and accepted. We acknowledge that Hcy levels could have been affected by MTX use and folic acid intake, though there was no direct association between MTX use and cIMT or plaques. Thirdly, the sample size was relatively small, however this was not a multicenter study, as patients and controls were recruited from the outpatient clinics of two tertiary hospitals.

In conclusion, there appear to be some significant associations between specific SNPs and the progression of atherosclerosis and CVD in RA. It remains unclear whether in RA these associations reflect a common genetic pathway implicated in the pathogenesis of both RA and atherosclerosis, or whether these genes, due to their genetic proximity or function, result- though gene-environment interaction, in the acceleration of CVD in RA.

## Methods

### Study population

In this prospective study, we enrolled 283 consecutive patients with RA (aged 60.8 ± 11.9 years, 16% men), who met the 1987 revised criteria of the American College of Rheumatology^99^ and attended the Rheumatology Outpatient Clinics of Laikon General Hospital and General Hospital of Athens “G. Gennimatas” and 595 healthy controls (HC) (aged 67.7 ± 12.4 years, 20% men). Our control group consisted of volunteering employees at Laikon Hospital, as well as of individuals referred to our laboratory for suspected arterial hypertension and/or for cardiovascular examination. Patients and HC were at least 18 years old, did not have history of clinical CVD, malignancy, chronic renal failure, or other concomitant chronic or acute inflammatory disease.

The study was approved by the Institutional Body Review and all subjects provided informed consent according to the Declaration of Helsinki.

### Clinical, laboratory and subclinical atherosclerosis assessment

All 283 RA patients and 280 HC were comprehensively studied for (a) preclinical atheromatosis defined by the presence of carotid and/or femoral artery plaques in the far and near wall of eight arterial sites (left and right common, internal carotid arteries and carotid bulb, and common femoral arteries)^3,100^ and (b) arterial hypertrophy of the common carotid arteries by IMT. Plaques were defined as local increase of the IMT of > 50% compared with the surrounding vessel wall, an IMT > 1.5 mm, or local thickening > 0.5 mm. All ultrasound measurements were performed using high-resolution B-mode ultrasound.

The presence of classical CV risk factors, as well as blood test measurements and other clinical parameters (DAS28) were identified from each patient’s file.

### Osteoprotegerin and MTHFR genotyping

Peripheral blood DNA samples from 262 RA patients and 234 HC were genotyped for the detection of the OPG rs2073618 variant. Additionally, 282 RA patients and 407 HC were studied for MTHFR A1298Cand C677T gene polymorphisms. Genomic DNA was extracted from blood samples, collected in EDTA tubes, using the Nucleospin Blood QuickPure kit (Macherey–Nagel GmbH & Co, Germany), according to the manufacturer’s instructions. DNA concentration was spectrophotometrically measured with Biospec-Nano (Shimadzu, Japan).

The rs2073618 polymorphism of the OPG gene and the A1298C (rs1801131) and C677T (rs1801133) polymorphisms of the MTHFR gene were analyzed by polymerase chain reaction (PCR) of genomic DNA, as previously described^40,101^. Briefly, the rs2073618 polymorphism of the OPG gene was analysed using TaqMan™ Allelic Discrimination assay according to the manufacturer’s instructions (Applied Biosystems, Foster City, CA, United States). The SNP ID is (C___1971047_40). PCRs were carried out using 30 ng of DNA with Genotyping Master Mix (Applied Biosystems) in a BIORAD IQ5 real-time PCR detection system (Bio-Rad, United States) for 35 cycles. Genotype quality assurance was assessed by random selection of 10% of DNA samples for re-genotyping, and the results were 100% concordant. The MTHFR rs1801133 polymorphism was analyzed by polymerase chain reaction‐restriction fragment length polymorphism (PCR‐RFLP) using the following primer pairs: 5′-TGAAGGAGAAGGTGTCTGCGGGA-3′ (forward) and 5′-AGGACGGTGCGGTGAGAGTG-3′ (reverse) generating a 198 bp product. PCR was carried out in a total volume of 15 μl with the Kapa Taq Ready Mix PCR Kit (Kapa Biosystems, Germany), containing 50 ng of genomic DNA and 0.2 μM of each primer in Eppendorf Mastercycler (Eppendorf, Germany). PCR amplification conditions were predenaturation at 95 °C for 30 s, annealing at 58 °C for 30 s and extension at 72 °C for 30 s, with a final extension step at 72 °C for 3 min. The PCR products were digested with restriction enzyme *Hinf* I (New England Biolabs) at 37 °C for 1 h and observed by 2, 5% agarose gel electrophoresis. The wild type homozygous (CC), heterozygous (CT) and mutant homozygous (TT) genotypes produce one band of 198 bp, three bands of 198, 175 and 23 bp and two bands of 175 and 23 bp respectively.

The c. 1298A>C (rs1801131) polymorphism was determined using the following primer pairs: 5′-CTTTGGGGAGCTGAAGGACTACTAC-3′ (forward) and 5′-CACTTTGTGACCATTCCGGTTTG-3′ (reverse), using the same PCR conditions that were used for the C. 677C>T mutation. The amplified fragment of 163 bp was digested for 1 h at 37 °C with 10 units of the restriction enzyme MboII (New England Biolabs). The digestion of the 1298AA genotype (normal) results in five fragments of 56, 31, 30, 28 and 18 bp, the 1298CC genotype (mutated) in four fragments of 84, 31, 30 and 18 bp, whereas the 1298AC genotype (heterozygous) in six fragments of 84, 56, 31, 30, 28 and 18 bp.

### Statistical analysis

Normality of sample distribution was examined by the Kolmogorov Smirnov test. Continuous variables are presented as mean (S.D.) when sample had a normal distribution, or median and 25th and75th percentile values [interquartile range (IQR)] for non-normally distributed samples. Categorical variables are presented as percentiles and are compared by Fisher’s exact tests. For normal or non-parametric distribution, continuous variables were compared by t-test or Mann Whitney test, respectively. Allele and genotype frequencies in RA patients and HC were determined for each SNP by SNPStats software^102^. Genotypic frequencies in control subjects for each SNP were tested for departure from Hardy-Weinbergequilibrium. The Akaike information criterion (AIC) were also determined in all SNPstats tests. The Akaike information criterion (AIC) were also determined in all SNPstats tests. Lower AIC values indicate a better-fit model. Genotype frequencies for each SNP of RA anti-CCP positive patients were compared to both anti-CCP negative patients and HC using theχ^2^ test. ORs and corresponding 95% CIs were estimated by unconditional logistic regression adjusting for the effects of age and gender. The five genetic models (codominant, dominant, recessive, overdominant and additive) were also determined^102^. To explore independent predictors for high IMT carotid scores, multivariate logistic regression models were implemented, taking into account CVD risk factors (BMI, smoking, hypertension, lipid levels). SPSS version 26 and Stata version 12 (StataCorp, College Station, TX, USA) were used for analyses and p < 0.05 was considered as the level of statistical significance in all cases.

### Ethics statement

The study was reviewed and approved by Laikon General Hospital of Athens and “G. Gennimatas” General Hospital of Athens Institutional Body Review. All subjects gave informed consent in accordance with the Declaration of Helsinki.

## Supplementary Information

Supplementary Tables.

## Data Availability

All data relevant to the study are included in the article or uploaded as supplementary information.

## References

[1] Pasceri V, Yeh ET (1999). A tale of two diseases: Atherosclerosis and rheumatoid arthritis. Circulation.

[2] Arida A, Protogerou AD, Kitas GD, Sfikakis PP (2018). Systemic inflammatory response and atherosclerosis: The paradigm of chronic inflammatory rheumatic diseases. Int. J. Mol. Sci..

[3] Protogerou A, Zampeli E, Tentolouris N, Makrilakis K, Kitas G, Sfikakis PP (2012). Subclinical femoral atheromatosis in rheumatoid arthritis: Comparable prevalence to diabetes mellitus in a case-control study. Ann. Rheum. Dis..

[4] López-Mejías R, Castañeda S, González-Juanatey C, Corrales A, Ferraz-Amaro I, Genre F, Remuzgo-Martínez S, Rodriguez-Rodriguez L, Blanco R, Llorca J, Martín J, González-Gay MA (2016). Cardiovascular risk assessment in patients with rheumatoid arthritis: The relevance of clinical, genetic and serological markers. Autoimmun. Rev..

[5] Lacey DL, Timms E, Tan HL, Kelley MJ, Dunstan CR, Burgess T, Elliott R, Colombero A, Elliott G, Scully S, Hsu H, Sullivan J, Hawkins N, Davy E, Capparelli C, Eli A, Qian YX, Kaufman S, Sarosi I, Shalhoub V, Senaldi G, Guo J, Delaney J, Boyle WJ (1998). Osteoprotegerin ligand is a cytokine that regulates osteoclast differentiation and activation. Cell.

[6] Simonet, W., Lacey, D., & Dunstan, C. Osteoprotegerin: a novel secreted protein involved in the regulation of bone density, Elsevier. (n.d.). 1997. https://www.sciencedirect.com/science/article/pii/S0092867400802093. Accessed 12 Mar 2021. 10.1016/s0092-8674(00)80209-3 9108485

[7] Rochette L, Meloux A, Rigal E, Zeller M, Cottin Y, Vergely C (2018). The role of osteoprotegerin in the crosstalk between vessels and bone: Its potential utility as a marker of cardiometabolic diseases. Pharmacol. Ther..

[8] Bucay, N., Sarosi, I., Dunstan, C.R., Morony, S., Tarpley, J., Capparelli, C., Scully, S., Tan, H.L., Xu, W., Lacey, D.L., Boyle, W.J., & Simonet, W.S. Osteoprotegerin-deficient mice develop early onset osteoporosis and arterial calcification (1998). www.genesdev.org. Accessed 12 March 2021. 10.1101/gad.12.9.1260 PMC316769 9573043

[9] Bennett BJ, Scatena M, Kirk EA, Rattazzi M, Varon RM, Averill M, Schwartz SM, Giachelli CM, Rosenfeld ME (2006). Osteoprotegerin inactivation accelerates advanced atherosclerotic lesion progression and calcification in older ApoE-/- mice. Arterioscler. Thromb. Vasc. Biol..

[10] Kiechl S, Schett G, Wenning G, Redlich K, Oberhollenzer M, Mayr A, Santer P, Smolen J, Poewe W, Willeit J (2004). Osteoprotegerin is a risk factor for progressive atherosclerosis and cardiovascular disease. Circulation.

[11] Wahlin, B., Ramnemark, A., Rantapää-Dahlqvist, S., Wållberg-Jonsson, S., & Södergren, A. Osteoprotegerin and osteocalcin are associated with atherosclerosis in patients with rheumatoid arthritis: a prospective cohort study. *Clin. Exp. Rheumatol.* (2021). http://www.ncbi.nlm.nih.gov/pubmed/33635235. Accessed 12 Mar 2021. 10.55563/clinexprheumatol/5p7mb4 33635235

[12] Jono S, Ikari Y, Shioi A, Mori K, Miki T, Hara K, Nishizawa Y (2002). Serum osteoprotegerin levels are associated with the presence and severity of coronary artery disease. Circulation.

[13] Straface G, Biscetti F, Pitocco D, Bertoletti G, Misuraca M, Vincenzoni C, Snider F, Arena V, Stigliano E, Angelini F, Iuliano L, Boccia S, De Waure C, Giacchi F, Ghirlanda G, Flex A (2011). Assessment of the genetic effects of polymorphisms in the osteoprotegerin gene, TNFRSF11B, on serum osteoprotegerin levels and carotid plaque vulnerability. Stroke.

[14] Biscetti F, Straface G, Giovannini S, Santoliquido A, Angelini F, Santoro L, Porreca CF, Pecorini G, Ghirlanda G, Flex A (2013). Association between TNFRSF11B gene polymorphisms and history of ischemic stroke in Italian diabetic patients. Hum. Genet..

[15] Strand M, Söderström I, Wiklund PG, Hallmans G, Weinehall L, Söderberg S, Olsson T (2007). Polymorphisms at the osteoprotegerin and interleukin-6 genes in relation to first-ever stroke. Cerebrovasc. Dis..

[16] Chen Y, Yang Y, Liu G (2016). Association between osteoprotegerin gene polymorphisms and rheumatoid arthritis susceptibility: A meta-analysis. Arch. Med. Res..

[17] Chung CP, Solus JF, Oeser A, Li C, Raggi P, Smith JR, Michael Stein C (2015). A variant in the osteoprotegerin gene is associated with coronary atherosclerosis in patients with rheumatoid arthritis: Results from a candidate gene study. Int. J. Mol. Sci..

[18] Genre F, López-Mejias R (2021). Osteoprotegerin CGA Haplotype Protection against Cerebrovascular Complications in Anti-CCP Negative Patients with Rheumatoid Arthritis. Rheum. Arthritis.

[19] Clarke R, Daly L, Robinson K, Naughten E, Cahalane S, Fowler B, Graham I (1991). Hyperhomocysteinemia: An independent risk factor for vascular disease. N. Engl. J. Med..

[20] Welch GN, Loscalzo J (1998). Homocysteine and Atherothrombosis. N. Engl. J. Med..

[21] Hoogeveen EK, Kostense PJ, Beks PJ, Mackaay AJC, Jakobs C, Bouter LM, Heine RJ, Stehouwer CDA (1998). Hyperhomocysteinemia is associated with an increased risk of cardiovascular disease, especially in non-insulin-dependent diabetes mellitus: A population-based study. Arterioscler. Thromb. Vasc. Biol..

[22] Lazzerini PE, Capecchi PL, Selvi E, Lorenzini S, Bisogno S, Galeazzi M, Laghi Pasini F (2007). Hyperhomocysteinemia: A cardiovascular risk factor in autoimmune diseases?. Lupus.

[23] Petri M, Roubenoff R, Dallal GE, Nadeau MR, Selhub J, Rosenberg IH (1996). Plasma homocysteine as a risk factor for atherothrombotic events in systemic lupus erythematosus. Lancet.

[24] Cisternas M, Gutiérrez MA, Klaassen J, Acosta AM, Jacobelli S (2002). Cardiovascular risk factors in Chilean patients with rheumatoid arthritis. J. Rheumatol..

[25] Antoniades C, Shirodaria C, Leeson P, Baarholm OA, Van-Assche T, Cunnington C, Pillai R, Ratnatunga C, Tousoulis D, Stefanadis C, Refsum H, Channon KM (2009). MTHFR 677 C>T polymorphism reveals functional importance for 5-methyltetrahydrofolate, not homocysteine, in regulation of vascular redox state and endothelial function in human atherosclerosis. Circulation.

[26] Kluijtmans LAJ, Van Den Heuvel LPWJ, Boers GHJ, Frosst P, Stevens EMB, Van Oost BA, Den Heijer M, Trijbels FJM, Rozen R, Blom HJ (1996). Molecular genetic analysis in mild hyperhomocysteinemia: A common mutation in the methylenetetrahydrofolate reductase gene is a genetic risk factor for cardiovascular disease. Am. J. Hum. Genet..

[27] Moll S, Varga EA (2015). Homocysteine and MTHFR mutations. Circulation.

[28] Abd El-Aziz TA, Mohamed RH (2017). Influence of MTHFR C677T gene polymorphism in the development of cardiovascular disease in Egyptian patients with rheumatoid arthritis. Gene.

[29] Davis LA, Cannon GW, Pointer LF, Haverhals LM, Wolff RK, Mikuls TR, Reimold AM, Kerr GS, Richards JS, Johnson DS, Valuck R, Prochazka A, Caplan L (2013). Cardiovascular events are not associated with MTHFR polymorphisms, but are associated with methotrexate use and traditional risk factors in US veterans with rheumatoid arthritis. J. Rheumatol..

[30] Solomon DH, Greenberg J, Curtis JR, Liu M, Farkouh ME, Tsao P, Kremer JM, Etzel CJ (2015). Derivation and internal validation of an expanded cardiovascular risk prediction score for rheumatoid arthritis: a Consortium of Rheumatology Researchers of North America Registry Study. Arthritis Rheumatol..

[31] Wang P, Li S, Liu LN, Lv TT, Li XM, Li XP, Pan HF (2017). Circulating osteoprotegerin levels are elevated in rheumatoid arthritis: A systematic review and meta-analysis. Clin. Rheumatol..

[32] Saidenberg-Kermanach N, Cohen-Solal M, Bessis N, De Vernejoul MC, Boissier MC (2004). Role for osteoprotegerin in rheumatoid inflammation. Jt. Bone Spine..

[33] Van Steenbergen HW, Van Der Helm-Van AHM (2016). Osteoprotegerin as biomarker for persistence of rheumatoid arthritis. Rheumatol. (United Kingdom).

[34] Yang H, Liu W, Zhou X, Rui H, Zhang H, Liu R (2019). The association between RANK, RANKL and OPG gene polymorphisms and the risk of rheumatoid arthritis: A case-controlled study and meta-analysis. Biosci. Rep..

[35] Ruyssen-Witrand A, Degboé Y, Cantagrel A, Nigon D, Lukas C, Scaramuzzino S, Allanore Y, Vittecoq O, Schaeverbeke T, Morel J, Sibilia J, Cambon-Thomsen A, Dieudé P, Constantin A (2016). Association between RANK, RANKL and OPG polymorphisms with ACPA and erosions in rheumatoid arthritis: results from a meta-analysis involving three French cohorts. RMD Open.

[36] Asanuma Y, Chung CP, Oeser A, Solus JF, Avalos I, Gebretsadik T, Shintani A, Raggi P, Sokka T, Pincus T, Stein CM (2007). Serum osteoprotegerin is increased and independently associated with coronary-artery atherosclerosis in patients with rheumatoid arthritis. Atherosclerosis.

[37] Dessein PH, Loṕez-Mejias R, Gonzaĺez-Juanatey C, Genre F, Miranda-Filloy JA, Llorca J, González-Gay MA (2014). Independent relationship of osteoprotegerin concentrations with endothelial activation and carotid atherosclerosis in patients with severe rheumatoid arthritis. J. Rheumatol..

[38] Lin JF, Wu S, Juang JMJ, Chiang FT, Hsu LA, Teng MS, Cheng ST, Huang HL, Ko YL (2019). Osteoprotegerin and osteopontin levels, but not gene polymorphisms, predict mortality in cardiovascular diseases. Biomark. Med..

[39] Song DH, Zhou PZ, Xiu XL, Zou GH, Sun YX, Song C (2016). Relationships of OPG genetic polymorphisms with susceptibility to cardiovascular disease: A meta-analysis. Med. Sci. Monit..

[40] Pérez-Hernández N, Posadas-Sánchez R, Vargas-Alarcón G, Cazarín-Santos BG, Miranda-Duarte A, Rodríguez-Pérez JM (2020). Genetic variants and haplotypes in OPG gene are associated with premature coronary artery disease and traditional cardiovascular risk factors in Mexican population: The GEA study. DNA Cell Biol..

[41] Klerk M, Verhoef P, Clarke R, Blom HJ, Kok FJ, Schouten EG, Abbate R, Marcucci R, Samani NJ, Anderson JL, Zebrack JS, Ardissino D, Merlini FM, Bonomi AB, Van Bockxmeer FM, Brownrigg L, Chambers J, Kooner JS, Genest J, Rozen R, Ferrer-Antunes C, Palmeiro A, Fernandez-Arcas N, Reyes-Engel A, Folsom AR, Fowkes FGR, Lee AJ, Gemmati D, Scapoli GL, Girelli D, Corrocher R, Gulec S, Hopkins PN, Inbal A, Selighson U, Kluijtmans LAJ, Jukema JW, Kozich V, Janosikova B, Ma J, Stampfer MJ, Malinow MR, Ashfield-Watt PAL, Clark ZE, Meisel C, Stangl K, Graham IM, Morita H, Nagai R, Nakai K, Yamakawa-Kobayashi K, Hamaguchi H, Gaziano M, Schwartz SM, Siscovick DS, Silberberg JS, Szczeklik A, Domagala Teresa B, Tanis BC, Rosendaal FM, Thogersen AM, Nilsson TK, Todesco L, Litynsky P, Tokgozoglu SL, Tsai MY, Hanson NQ, Rimm EB, Verhoeff BJ, Trip MD (2002). MTHFR 677C→T polymorphism and risk of coronary heart disease: A meta-analysis. J. Am. Med. Assoc..

[42] Luo Z, Lu Z, Muhammad I, Chen Y, Chen Q, Zhang J, Song Y (2018). Associations of the MTHFR rs1801133 polymorphism with coronary artery disease and lipid levels: A systematic review and updated meta-analysis. Lipids Health Dis..

[43] Lievers KJ, Boers GH, Verhoef P, Heijer M, Kluijtmans LA, Put NM, Trijbels FJ, Blom HJ (2001). A second common variant in the methylenetetrahydrofolate reductase (MTHFR) gene and its relationship to MTHFR enzyme activity, homocysteine, and cardiovascular disease risk. J. Mol. Med..

[44] Giannelou M, Nezos A, Fragkioudaki S, Kasara D, Maselou K, Drakoulis N, Ioakeimidis D, Moutsopoulos HM, Mavragani CP (2018). Contribution of MTHFR gene variants in lupus related subclinical atherosclerosis. Clin. Immunol..

[45] Hayta E, Hizmetli S, Atalar MH, Çinar Z (2015). Association of plasma homocysteine level and carotid intima-media thickness in rheumatoid arthritis patients receiving methotrexate. Arch. Rheumatol..

[46] Głuszek J, Wierzowiecka M, Niklas K, Niklas A (2020). The importance of homocysteine in the development of cardiovascular complications in patients with rheumatoid arthritis. Reumatologia.

[47] Fujimaki C, Hayashi H, Tsuboi S, Matsuyama T, Kosuge K, Yamada H, Inoue K, Itoh K (2009). Plasma total homocysteine level and methylenetetrahydrofolate reductase 677C>T genetic polymorphism in Japanese patients with rheumatoid arthritis. Biomarkers.

[48] Palomino-Morales R, Gonzalez-Juanatey C, Vazquez-Rodriguez TR, Rodriguez L, Miranda-Filloy JA, Fernandez-Gutierrez B, Llorca J, Martin J, Gonzalez-Gay MA (2010). A1298C polymorphism in the MTHFR gene predisposes to cardiovascular risk in rheumatoid arthritis. Arthritis Res. Ther..

[49] Remuzgo-Martínez S, Genre F, López-Mejías R, Ubilla B, Mijares V, Pina T, Corrales A, Blanco R, Martín J, Llorca J, González-Gay MA (2016). Decreased expression of the methylene tetrahydrofolate reductase (MTHFR) gene in patients with rheumatoid arthritis. Clin. Exp. Rheumatol..

[50] Bhattacharyya T, Nicholls SJ, Topol EJ, Zhang R, Yang X, Schmitt D, Fu X, Shao M, Brennan DM, Ellis SG, Brennan ML, Allayee H, Lusis AJ, Hazen SL (2008). Relationship of paraoxonase 1 (PON1) gene polymorphisms and functional activity with systemic oxidative stress and cardiovascular risk. JAMA J. Am. Med. Assoc..

[51] Zaragoza-García O, Guzmán-Guzmán IP, Moreno-Godínez ME, Navarro-Zarza JE, Antonio-Vejar V, Ramírez M, Parra-Rojas I (2021). PON-1 haplotype (-108C>T, L55M, and Q192R) modulates the serum levels and activity PONase promoting an atherogenic lipid profile in rheumatoid arthritis patients. Clin. Rheumatol..

[52] Navarro-Millán I, Charles-Schoeman C, Yang S, Bathon JM, Bridges SL, Chen L, Cofield SS, Dell’Italia LJ, Moreland LW, O’Dell JR, Paulus HE, Curtis JR (2013). Changes in lipoproteins associated with methotrexate or combination therapy in early rheumatoid arthritis: Results from the treatment of early rheumatoid arthritis trial. Arthritis Rheum..

[53] Rodríguez-Carrio J, López-Mejías R, Alperi-López M, López P, Ballina-García FJ, González-Gay M, Suárez A (2016). Paraoxonase 1 activity is modulated by the rs662 polymorphism and IgG anti-high-density lipoprotein antibodies in patients with rheumatoid arthritis: Potential implications for cardiovascular disease. Arthritis Rheumatol..

[54] Mucientes A, Fernández-Gutiérrez B, Herranz E, Rodriguez-Rodriguez L, Varadé J, Urcelay E, Lamas JR (2019). Functional implications of single nucleotide polymorphisms rs662 and rs854860 on the antioxidative activity of paraoxonase1 (PON1) in patients with rheumatoid arthritis. Clin. Rheumatol..

[55] Atwa ET, Hussin AG, Mohamed MR, Pasha HF, Hammad M (2020). Carotid plaques in adult rheumatoid arthritis patients; association with paroxonase 1 enzymatic activity and Q192R paroxonase 1 gene polymorphism. Mol. Biol. Rep..

[56] Khoja SO, El Miedany Y, Iyer AP, Bahlas SM, Balamash KS, Elshal MF (2017). Association of paraoxonase 1 polymorphism and serum 25-hydroxyvitamin D with the risk of cardiovascular disease in patients with rheumatoid arthritis. Clin. Lab..

[57] Charles-Schoeman C, Lee YY, Shahbazian A, Gorn AH, Fitzgerald J, Ranganath VK, Taylor M, Ragavendra N, McMahon M, Elashoff D, Reddy ST (2013). Association of paraoxonase 1 gene polymorphism and enzyme activity with carotid plaque in rheumatoid arthritis. Arthritis Rheum..

[58] Eichner JE (2002). Apolipoprotein E polymorphism and cardiovascular disease: A HuGE review. Am. J. Epidemiol..

[59] Toms TE, Smith JP, Panoulas VF, Blackmore H, Douglas KMJ, Kitas GD (2012). Apolipoprotein E gene polymorphisms are strong predictors of inflammation and dyslipidemia in rheumatoid arthritis. J. Rheumatol..

[60] Maehlen MT, Provan SA, de Rooy DPC, van der Helm-van Mil AHM, Krabben A, Saxne T, Lindqvist E, Semb AG, Uhlig T, van der Heijde D, Mero IL, Olsen IC, Kvien TK, Lie BA (2013). Associations between APOE genotypes and disease susceptibility, joint damage and lipid levels in patients with rheumatoid arthritis. PLoS ONE.

[61] Chen YM, Chen PK, Chang CK, Lin CC, Chen HH, Lan JL, Chang SH, Chen DY (2020). Association of apolipoprotein e polymorphism with adipokines and cardiovascular disease risk in rheumatoid arthritis patients. Life..

[62] Colombo MG, Paradossi U, Andreassi MG, Botto N, Manfredi S, Masetti S, Biagini A, Clerico A (2003). Endothelial nitric oxide synthase gene polymorphisms and risk of coronary artery disease. Clin. Chem..

[63] Casas JP, Cavalleri GL, Bautista LE, Smeeth L, Humphries SE, Hingorani AD (2006). By the Johns Hopkins Bloomberg School of Public Health All rights reserved. Am. J. Epidemiol..

[64] Melchers I, Blaschke S, Hecker M, Cattaruzza M (2006). The ^-786^ C/T single-nucleotide polymorphism in the promoter of the gene for endothelial nitric oxide synthase: Insensitivity to physiologic stimuli as a risk factor for rheumatoid arthritis. Arthritis Rheum..

[65] Pascual M, López-Nevot MA, Cáliz R, Koeleman BPC, Balsa A, Pascual-Salcedo D, Martín J (2002). Genetic determinants of rheumatoid arthritis: The inducible nitric oxide synthase (NOS2) gene promoter polymorphism. Genes Immun..

[66] Gonzalez-Gay MA (2004). Inducible but not endothelial nitric oxide synthase polymorphism is associated with susceptibility to rheumatoid arthritis in northwest Spain. Rheumatology.

[67] Brenol CV, Chies JAB, Brenol JCT, Xavier RM (2010). Role of endothelial nitric oxide synthase (eNOS) polymorphisms in cardiovascular disease and rheumatoid arthritis. Clin. Exp. Rheumatol..

[68] Gonzalez-Gay, M.A., Llorca, J., Palomino-Morales, R., Gomez-Acebo, I., Gonzalez-Juanatey, C., Martin, J., Gonzalez-Gay, M.A., Gonzalez-Juanatey, C., Llorca, J., Gomez-Acebo, I., Palomino-Morales, R., & Martin, J. Influence of nitric oxide synthase gene polymorphisms on the risk of cardiovascular events in rheumatoid arthritis. https://www.clinexprheumatol.org/article.asp?a=3579. Accessed 1 Apr 2021. 19327239

[69] Luo Y, Wang Y, Luo W (2020). C allele of -786 T>C polymorphism in the promoter region of endothelial nitric oxide synthase is responsible for endothelial dysfunction in the patients with rheumatoid arthritis. J. Cell. Biochem..

[70] Dimitroulas T, Kitas GD (2019). Genetic regulation of dimethylarginines and endothelial dysfunction in rheumatoid arthritis. Amino Acids.

[71] Dimitroulas T, Hodson J, Panoulas VF, Sandoo A, Smith J, Kitas G (2017). Genetic variations in the alanine–glyoxylate aminotransferase 2 (AGXT2) gene and dimethylarginines levels in rheumatoid arthritis. Amino Acids.

[72] Leonard D, Svenungsson E, Dahlqvist J, Alexsson A, Ärlestig L, Taylor KE, Sandling JK, Bengtsson C, Frodlund M, Jönsen A, Eketjäll S, Jensen-Urstad K, Gunnarsson I, Sjöwall C, Bengtsson AA, Eloranta ML, Syvänen AC, Rantapää-Dahlqvist S, Criswell LA, Rönnblom L (2018). Novel gene variants associated with cardiovascular disease in systemic lupus erythematosus and rheumatoid arthritis. Ann. Rheum. Dis..

[73] Damen MSMA, Agca R, Holewijn S, De Graaf J, Dos Santos JC, Van Riel PL, Fransen J, Coenen MJH, Nurmohamed MT, Netea MG, Dinarello CA, Joosten LAB, Heinhuis B, Popa CD (2017). IL-32 promoter SNP rs4786370 predisposes to modified lipoprotein profiles in patients with rheumatoid arthritis. Sci. Rep..

[74] Farias TDJ, Do Cantoa LM, Medeiros MD, Sereia AFR (2013). Ausência de associação entre os polimorfismos do gene interleucina-18 e artrite reumatoide. Rev. Bras. Reumatol..

[75] Amaya-Amaya J, Rojas-Villarraga A, Molano-Gonzalez N, Montoya-Sánchez L, Nath SK, Anaya JM (2015). GDF15(MIC1) H6D polymorphism does not influence cardiovascular disease in a Latin American population with rheumatoid arthritis. J. Immunol. Res..

[76] García-Bermúdez M, López-Mejías R, Genre F, Castañeda S, Llorca J, González-Juanatey C, Corrales A, Ubilla B, Miranda-Filloy JA, Pina T, Gómez-Vaquero C, Rodríguez-Rodríguez L, Fernández-Gutiérrez B, Balsa A, Pascual-Salcedo D, López-Longo FJ, Carreira P, Blanco R, Martín J, González-Gay MA (2014). Interferon regulatory factor 5 genetic variants are associated with cardiovascular disease in patients with rheumatoid arthritis. Arthritis Res. Ther..

[77] Agca R, van Sijl AM, Vosslamber S, Voskuyl AE, Verweij CL, Nurmohamed MT (2021). Interferon regulatory factor 5 gene variants rs2004640 and rs4728142 are associated with carotid intima media thickness but not with cardiovascular events in rheumatoid arthritis. Clin. Exp. Rheumatol..

[78] Danesh J, Wheeler JG, Hirschfield GM, Eda S, Eiriksdottir G, Rumley A, Lowe GDO, Pepys MB, Gudnason V (2004). C-reactive protein and other circulating markers of inflammation in the prediction of coronary heart disease. N. Engl. J. Med..

[79] Lagrand WK, Visser CA, Hermens WT, Niessen HWM, Verheugt FWA, Wolbink GJ, Hack CE (1999). C-reactive protein as a cardiovascular risk factor more than an epiphenomenon?. Circulation.

[80] Miller DT, Zee RYL, Suk Danik J, Kozlowski P, Chasman DI, Lazarus R, Cook NR, Ridker PM, Kwiatkowski DJ (2005). Association of common *CRP* gene variants with CRP levels and cardiovascular events. Ann. Hum. Genet..

[81] Del Rincón I, Williams K, Stern MP, Freeman GL (2003). Association between carotid atherosclerosis and markers of inflammation in rheumatoid arthritis patients and healthy subjects. Arthritis Rheum..

[82] Gonzalez-Gay MA, Gonzalez-Juanatey C, Piñeiro A, Garcia-Porrua C, Testa A, Llorca J (2005). High-grade C-reactive protein elevation correlates with accelerated atherogenesis in patients with rheumatoid arthritis. J. Rheumatol..

[83] Lange LA, Carlson CS, Hindorff LA, Lange EM, Walston J, Durda JP, Cushman M, Bis JC, Zeng D, Lin D, Kuller LH, Nickerson DA, Psaty BM, Tracy RP, Reiner AP (2006). Association of polymorphisms in the CRP gene with circulating C-reactive protein levels and cardiovascular events. J. Am. Med. Assoc..

[84] Hage FG, Szalai AJ (2007). C-reactive protein gene polymorphisms, C-reactive protein blood levels, and cardiovascular disease risk. J. Am. Coll. Cardiol..

[85] Ibrahim I, Plant D, Lunt M, Verstappen S, Flynn E, Symmons D, Barton A (2013). Investigation of C reactive protein gene polymorphisms as predictors of cardiovascular mortality in inflammatory polyarthritis: Results from the Norfolk Arthritis Register. Ann. Rheum. Dis..

[86] López-Mejiás R, Genre F, Remuzgo-Martínez S, González-Juanatey C, Robustillo-Villarino M, Llorca J, Corrales A, Vicente E, Miranda-Filloy JA, Magro C, Tejera-Segura B, Ramírez Huaranga MA, Pina T, Blanco R, Alegre-Sancho JJ, Raya E, Mijares V, Ubilla B, Mínguez Sánchez MD, Gómez-Vaquero C, Balsa A, Pascual-Salcedo D, López-Longo FJ, Carreira P, González-Álvaro I, Rodríguez-Rodríguez L, Fernández-Gutiérrez B, Ferraz-Amaro I, Castañeda S, Martín J, González-Gay MA (2016). Influence of elevated-CRP level-related polymorphisms in non-rheumatic Caucasians on the risk of subclinical atherosclerosis and cardiovascular disease in rheumatoid arthritis. Sci. Rep..

[87] Kastbom A, Ärlestig L, Rantapää-Dahlqvist S (2015). Genetic variants of the NLRP3 inflammasome are associated with stroke in patients with rheumatoid arthritis. J. Rheumatol..

[88] López-Mejías R, Carmona FD, Genre F, Remuzgo-Martínez S, González-Juanatey C, Corrales A, Vicente EF, Pulito-Cueto V, Miranda-Filloy JA, Ramírez Huaranga MA, Blanco R, Robustillo-Villarino M, Rodríguez-Carrio J, Alperi-López M, Alegre-Sancho JJ, Mijares V, Lera-Gómez L, Pérez-Pampín E, González A, Ortega-Castro R, López-Pedrera C, García Vivar ML, Gómez-Arango C, Raya E, Narvaez J, Balsa A, López-Longo FJ, Carreira P, González-Álvaro I, Rodríguez-Rodríguez L, Fernández-Gutiérrez B, Ferraz-Amaro I, Gualillo O, Castañeda S, Martín J, Llorca J, González-Gay MA (2019). Identification of a 3′-untranslated genetic variant of RARB associated with carotid intima-media thickness in rheumatoid arthritis: A genome-wide association study. Arthritis Rheumatol..

[89] Arya R, Escalante A, Farook VS, Restrepo JF, Battafarano DF, Almeida M, Kos MZ, Fourcaudot MJ, Mummidi S, Kumar S, Curran JE, Jenkinson CP, Blangero J, Duggirala R, del Rincon I (2018). A genetic association study of carotid intima-media thickness (CIMT) and plaque in Mexican Americans and European Americans with rheumatoid arthritis. Atherosclerosis.

[90] Pamuk ON, Tozkir H, Uyanik MS, Gurkan H, Saritas F, Duymaz J, Donmez S, Yazar M, Pamuk GE (2014). PECAM-1 gene polymorphisms and soluble PECAM-1 level in rheumatoid arthritis and systemic lupus erythematosus patients: Any link with clinical atherosclerotic events?. Clin. Rheumatol..

[91] Menghini R, Campia U, Tesauro M, Marino A, Rovella V, Rodia G, Schinzari F, Tolusso B, Di Daniele N, Federici M, Zoli A, Ferraccioli G, Cardillo C (2014). Toll-like receptor 4 mediates endothelial cell activation through NF-κB but is not associated with endothelial dysfunction in patients with rheumatoid arthritis. PLoS ONE.

[92] García-Bermúdez M, López-Mejías R, Genre F, Castañeda S, González-Juanatey C, Llorca J, Corrales A, Miranda-Filloy JA, Pina T, Gómez-Vaquero C, Rodríguez-Rodríguez L, Fernández-Gutiérrez B, Pascual-Salcedo D, Balsa A, López-Longo FJ, Carreira P, Blanco R, González-Álvaro I, Martín J, González-Gay MA (2013). Single-nucleotide polymorphisms at the 9p213 genomic region not associated with the risk of cardiovascular disease in patients with rheumatoid arthritis. Tissue Antigens.

[93] Davis LA, Whitfield E, Cannon GW, Wolff RK, Johnson DS, Reimold AM, Kerr GS, Richards JS, Mikuls TR, Caplan L (2014). Association of rheumatoid arthritis susceptibility gene with lipid profiles in patients with rheumatoid arthritis. Rheumatol. (United Kingdom).

[94] Park YJ, Yoo SA, Choi S, Yoo HS, Yoon HS, Cho CS, Yoo KD, Kim WU (2013). Association of polymorphisms modulating low-density lipoprotein cholesterol with susceptibility, severity, and progression of rheumatoid arthritis. J. Rheumatol..

[95] Panoulas VF, Douglas KMJ, Smith JP, Metsios GS, Elisaf MS, Nightingale P, Kitas GD (2009). Galectin-2 (LGALS2) 3279C/T polymorphism may be independently associated with diastolic blood pressure in patients with rheumatoid arthritis. Clin. Exp. Hypertens..

[96] Panoulas VF, Douglas KMJ, Smith JP, Stavropoulos-Kalinoglou A, Metsios GS, Nightingale P, Kitas GD (2009). Transforming growth factor-β1 869T/C, but not interleukin-6 -174G/ C, polymorphism associates with hypertension in rheumatoid arthritis. Rheumatology.

[97] Panoulas VF, Douglas KMJ, Smith JP, Taffe P, Stavropoulos-Kalinoglou A, Toms TE, Elisaf MS, Nightingale P, Kitas GD (2008). Polymorphisms of the endothelin-1 gene associate with hypertension in patients with rheumatoid arthritis. Endothel. J. Endothel Cell Res..

[98] Repeated cross-sectional study (2018). L.R. Baghdadi, R.J. Woodman, E. Michael Shanahan, M.D. Wiese, A.A. Mangoni, Genetic polymorphism of the methotrexate transporter ABCG2, blood pressure and markers of arterial function in patients with rheumatoid arthritis. Pharmgenomics. Pers. Med..

[99] Arnett FC, Edworthy SM, Bloch DA, Mcshane DJ, Fries JF, Cooper NS, Healey LA, Kaplan SR, Liang MH, Luthra HS, Medsger TA, Mitchell DM, Neustadt DH, Pinals RS, Schaller JG, Sharp JT, Wilder RL, Hunder GG (1988). Revised criteria for the classification of rheumatoid arthritis. Arthritis Rheum..

[100] Protogerou AD, Fransen J, Zampeli E, Argyris AA, Aissopou E, Arida A, Konstantonis GD, Tentolouris N, Makrilakis K, Psichogiou M, Daikos G, Kitas GD, Sfikakis PP (2015). The additive value of femoral ultrasound for subclinical atherosclerosis assessment in a single center cohort of 962 adults, including high risk patients with rheumatoid arthritis, Human Immunodeficiency Virus infection and Type 2 Diabetes Mellitus. PLoS ONE.

[101] Fragkioudaki S, Nezos A, Souliotis VL, Chatziandreou I, Saetta AA, Drakoulis N, Tzioufas AG, Voulgarelis M, Sfikakis PP, Koutsilieris M, Crow MK, Moutsopoulos HM, Mavragani CP (2017). MTHFR gene variants and non-MALT lymphoma development in primary Sjogren’s syndrome. Sci. Rep..

[102] Solé X, Guinó E, Valls J, Iniesta R, Moreno V (2006). SNPStats: A web tool for the analysis of association studies. Bioinformatics.

