# Supplementary material for: Osteoprotegerin and MTHFR gene variations in rheumatoid arthritis: association with disease susceptibility and markers of subclinical atherosclerosis
Source: Sci Rep. 2022 Jun 9;12:9534. doi: 10.1038/s41598-022-13265-3 (PMC9184606; doi:10.1038/s41598-022-13265-3)
Supplement: Supplementary file 1 — Supplementary Tables. [file 41598_2022_13265_MOESM1_ESM.docx]

**Supplementary Tables**

**Suppl Table 1.** Demographics, RA disease-related characteristics, clinical characteristics and subclinical cardiovascular (CVD) indices of 262 RA patients and 234 healthy controls (HC) analyzed for the rs2073618 polymorphism of the osteoprotegerin (OPG) gene. **bDMARDS: biological DMARDS**

|  | | **HC (n=234)**  **n=244** | **RA (n=262)** |
| --- | --- | --- | --- |
| **Age (Mean± SD)** | | 56.5±13.6 | 60.8±11.8 |
| **Gender (male)(%)** | | 50.9 | 16.0 |
| **BMI (kgs/m^2^)** | | 28.0±4.9 | 28.1±5.4 |
| **Disease duration** | | -- | 13.6±10.1 |
| **RF positive (%) (n=241)** | | -- | 61.4 |
| **Anti-CCP positive (%) (n=239)** | | -- | 59 |
| **ESR** | | -- | 25.1±21.2 |
| **DAS 28** | | -- | 3.3±1.4 |
| **Diabetes (%)** | | 0.4 | 6.5 |
| **Hypertension (%)** | | 59.4 | 44.1 |
| **Current Smokers (%)** | | 35.0 | 26.8 |
| **Total Cholesterol (mg/dl)** | | 198.3±36.3 | 203.0±36.8 |
| **LDL (mg/dl)** | | 122.7±30.5 | 120.5±30.6 |
| **Plaques (%)** | **Carotid and/or femoral** | 62.0 | 68.2 |
|  | **Carotid** | 47.0 | 58.9 |
|  | **Femoral** | 48.7 | 54.4 |
| **Carotid IMT (Mean± SD)**  **RCCA** | | 0.733±0.145 | 0.840±0.276 |
| **Methotrexate use (%)** | | -- | 53.1 |
| **bDMARDS use (%)** | | -- | 38.5 |

**Suppl Table 2.** Demographics, rheumatoid arthritis (RA) disease-related characteristics, clinical features and subclinical CVD indices of 282 RA patients and 46 healthy controls (HC) analyzed for the A1298C (rs1801131) and C677T (rs1801133)  polymorphisms of the MTHFR gene. **bDMARDS: biological DMARDS**

|  | | **HC (n=407)**  **n=244** | **RA (n=282)** |
| --- | --- | --- | --- |
| **Age (Mean± SD)** | | 55.2±17.8 | 60.9±11.8 |
| **Gender (male)(%)** | | 20.9 | 16.7 |
|  | | **HC (n=46)**  **n=244** |  |
| **BMI (kgs/m^2^)** | | 28.8±4.8 | 28.1±5.5 |
| **Disease duration** | | -- | 13.7±10.2 |
| **RF positive (%) (n=241)** | | -- | 62.1 |
| **Anti-CCP positive (%) (n=239)** | | -- | 58.9 |
| **ESR** | | -- | 26.1±22 |
| **DAS 28** | | -- | 3.4±1.4 |
| **Diabetes (%)** | | 0 | 7.1 |
| **Hypertension (%)** | | 63.0 | 43.6 |
| **Current Smokers (%)** | | 30.4 | 26.6 |
| **Total Cholesterol (mg/dl)** | | 195.2±39.4 | 202.7±36.3 |
| **LDL (mg/dl)** | | 120.5±34.1 | 120.4±30.4 |
| **Plaques (%)** | **Carotid and/or femoral** | 52.2 | 69.5 |
|  | **Carotid** | 39.1 | 61 |
|  | **Femoral** | 43.5 | 55.4 |
| **Carotid IMT (Mean± SD)**  **RCCA** | | 0.749±0.131 | 0.862±0.328 |
| **Methotrexate use (%)** | | -- | 53.5 |
| **bDMARDS use (%)** | | -- | 38.3 |

**Suppl Table 3.** Prevalence of the OPG rs2073618 and the MTHFR rs1801133 and rs1801131 SNP genotypes in **RA RF negative patients and HC**, adjusted by gender and age. Hardy-Weinberg equilibrium for control group p-values: 0.29, 0.76 and 0.55, for each SNP, respectively. Genotypes, OR and p-value for the five genetic models (codominant, dominant, recessive, overdominant and additive) were estimated with SNPstats software (statistically significant if p < 0.05). AIC lowest value for OPG rs2073618 SNP was in the recessive model: 393.6, for both MTHFR SNPs were in the recessive model, for the rs1801133 SNP:496.3 and for the rs1801131 SNP:502.8. OPG: osteoprotegerin, MTHFR: methylene tetrahydrofolate reductase, OR: odds ratio, SNP: single nucleotide polymorphism, vs: versus, RA: rheumatoid arthritis, HC: healthy controls, RF: rheumatoid factor, HAPMAP: haplotype map, AIC: Akaike information criterion.

| **SNPs** | **Genotype** | **HAPMAP**  **(European) Database (%)** | **HC n (%)** | **RA RF negative n (%)** | **OR codominant [95%CI]** | **p-value** | **ORdominant[95%CI]** | **p-value** | **OR recessive**  **[95%CI]** | **p-value** | **OR overdominant [95%CI]** | **p-value** | **OR logadditive**  **[95%CI]** | **p-value** |
| --- | --- | --- | --- | --- | --- | --- | --- | --- | --- | --- | --- | --- | --- | --- |
| **OPG rs2073618** |  |  |  |  | **CC vs CG vs GG** |  | **CC vs (CG/GG)** |  | **(CC/CG)**  **vs GG** |  | **CG vs (CC/GG)** |  |  |  |
|  | **CC** | 29 | 78 (33.3) | 31 (33.3) | 1.00 | 0.56 | 0.90  (0.52-1.57) | 0.72 | 1.28  (0.70-2.34) | 0.42 | 0.76  (0.45-1.29) | 0.31 | 1.04  (0.73-1.48) | 0.82 |
|  | **CG** | 53 | 107 (45.7) | 38 (40.9) | 0.80 (0.44-1.46) |  |  |  |  |  |  |  |  |  |
|  | **GG** | 18 | 49 (20.9) | 24 (25.8) | 1.13 (0.57-2.26) |  |  |  |  |  |  |  |  |  |
| **MTHFR rs1801133** |  |  |  |  | **CC vs CT vs TT** |  | **CC vs (CT/TT)** |  | **(CC/CT)**  **vs TT** |  | **CT vs (CC/TT)** |  |  |  |
|  | **CC** | 46.9 | 141 (34.6) | 42 (42.4) | 1.00 | 0.36 | 0.72  (0.46-1.14) | 0.16 | 0.94  (0.51-1.72) | 0.84 | 0.76  (0.48-1.19) | 0.22 | 0.84  (0.61-1.16) | 0.28 |
|  | **CT** | 44.2 | 200 (49.1) | 41 (41.4) | 0.70 (0.43-1.14) |  |  |  |  |  |  |  |  |  |
|  | **TT** | 8.8 | 66 (16.2) | 16 (16.2) | 0.78 (0.40-1.49) |  |  |  |  |  |  |  |  |  |
| **MTHFR rs1801131** |  |  |  |  | **AA vs AC vs CC** |  | **AA vs (AC/CC)** |  | **(AA/AC)**  **vs CC** |  | **AC vs (AA/CC)** |  |  |  |
|  | **AA** | 43.4 | 198 (48.6) | 44 (44.4) | 1.00 | 0.33 | 1.22  (0.78-1.91) | 0.38 | 1.70  (0.83-3.47) | 0.16 | 1.01  (0.65-1.59) | 0.95 | 1.26  (0.90-1.77) | 0.19 |
|  | **AC** | 45.1 | 176 (43.2) | 43 (43.4) | 1.12 (0.70-1.80) |  |  |  |  |  |  |  |  |  |
|  | **CC** | 11.5 | 33 (8.1) | 12 (12.1) | 1.79 (0.85-3.80) |  |  |  |  |  |  |  |  |  |

**Suppl Table 4.** Prevalence of the OPG rs2073618 and the MTHFR rs1801133 and rs1801131 SNP genotypes in **anti-CCP negative patients and HC**, adjusted by gender and age. Hardy-Weinberg equilibrium for control group p-values: 0.29, 0.76 and 0.55, respectively. Genotypes, OR and p-value for the five genetic models (codominant, dominant, recessive, overdominant and additive) were estimated with SNPstats software (statistically significant if p < 0.05). ). AIC lowest value for OPG rs2073618 SNP was in the overdominant model: 362.6, for both MTHFR SNPs were in the recessive model, rs1801133 SNP:521.0 and rs1801131 SNP:521.1. OPG: osteoprotegerin, MTHFR: methylene tetrahydrofolate reductase, OR: odds ratio, SNP: single nucleotide polymorphism, vs: versus, RA: rheumatoid arthritis, HC: healthy controls, anti-CCP: anti- cyclic citrullinated peptide, HAPMAP: haplotype map, AIC: Akaike information criterion.

| **SNPs** | **Genotype** | **HAPMAP (European) Database (%)** | **HC**  **n(%)** | **RA anti-CCP negative**  **n(%)** | **OR**  **codominant**  **model**  **[95%CI]** | **p-value** | **OR**  **dominant**  **model**  **[95%CI]** | **p-value** | **OR**  **recessive**  **model**  **[95%CI]** | **p-value** | **OR**  **overdominant**  **model**  **[95%CI]** | **p-value** | **OR logadditive**  **model**  **[95%CI]** | **p-value** |
| --- | --- | --- | --- | --- | --- | --- | --- | --- | --- | --- | --- | --- | --- | --- |
| **OPG rs2073618** |  |  |  |  | **CC vs CG vs GG** |  | **CC vs (CG/GG)** |  | **(CC/CG) vs GG** |  | **CG vs (CC/GG)** |  |  |  |
|  | **CC** | 29 | 78 (33.3) | 36 (36.7) | 1.00 | 0.037 | 0.77  (0.46-1.31) | 0.34 | 1.66  (0.94-2.93) | 0.08 | **0.52**  **(0.30-0.88)** | **0.013** | 1.07  (0.77-1.49) | 0.68 |
|  | **CG** | 53 | 107 (45.7) | 31 (31.6) | 0.56(0.31-1.03) |  |  |  |  |  |  |  |  |  |
|  | **GG** | 18 | 49 (20.9) | 31 (31.6) | 1.23(0.65-2.34) |  |  |  |  |  |  |  |  |  |
| **MTHFR rs1801133**  **(C677T)** |  |  |  |  | **CC vs CT vs TT** |  | **CC vs (CT/TT)** |  | **(CC/CT) vs TT** |  | **CT vs (CC/TT)** |  |  |  |
|  | **CC** | 46.9 | 141 (34.6) | 38 (35.5) | 1.00 | 0.54 | 0.92  (0.58-1.45) | 0.72 | 0.71  (0.38-1.33) | 0.27 | 1.10  (0.72-1.70) | 0.65 | 0.87  (0.64-1.20) | 0.41 |
|  | **CT** | 44.2 | 200 (49.1) | 55 (51.4) | 0.99(0.62-1.60) |  |  |  |  |  |  |  |  |  |
|  | **TT** | 8.8 | 66 (16.2) | 14 (13.1) | 0.71(0.35-1.41) |  |  |  |  |  |  |  |  |  |
| **MTHFR rs1801131**  **(A1298C)** |  |  |  |  | **AA vs AC vs CC** |  | **AA vs (AC/CC)** |  | **(AA/AC) vs CC** |  | **AC vs (AA/CC)** |  |  |  |
|  | **AA** | 43.4 | 198 (48.6) | 47 (43.9) | 1.00 | 0.44 | 1.25  (0.81-1.93) | 0.31 | 1.50  (0.72-3.12) | 0.29 | 1.10  (0.71-1.69) | 0.67 | 1.24  (0.89-1.74) | 0.21 |
|  | **AC** | 45.1 | 176 (43.2) | 49 (45.8) | 1.19(0.75-1.87) |  |  |  |  |  |  |  |  |  |
|  | **CC** | 11.5 | 33 (8.1) | 11 (10.3) | 1.63(0.76-3.52) |  |  |  |  |  |  |  |  |  |

**Supplementary Table 5.** Prevalence of the OPG rs2073618 and the MTHFR rs1801133 and rs1801131 SNP genotypes in RA anti-CCP positive and /or RF positive patients (seropositive RA) and HC, adjusted by gender and age. Hardy-Weinberg equilibrium for control group p-values: 0.2, 0.76 and 0.55, for each SNP, respectively. Genotypes, OR and p-value for the five genetic models (codominant, dominant, recessive, overdominant and additive) were estimated with SNPstats software (statistically significant if p < 0.05). AIC lowest value for OPG rs2073618 SNP was in the overdominant model: 490.0, for MTHFR rs1801133 was in the additive model:729.3 and, and for the rs1801131 SNP was in the recessive model:727.3. OPG: osteoprotegerin, MTHFR: methylene tetrahydrofolate reductase, OR: odds ratio, SNP: single nucleotide polymorphism, VS: versus, RA: rheumatoid arthritis, HC: healthy controls, HAPMAP: haplotype map.AIC:Akaike information criterion.

| **SNPs** | **Genotype** | **HAPMAP**  **(European) Database (%)** | **HC**  **n (%)** | **Seropositive RA**  **n (%)** | **OR codominant**  **model**  **[95%CI]** | **p-value** | **OR dominant**  **model**  **[95%CI]** | **p-value** | **OR recessive**  **model**  **[95%CI]** | **p-value** | **OR over dominant**  **model**  **[95%CI]** | **p-value** | **OR log**  **additive**  **model**  **[95%CI]** | **p-value** |
| --- | --- | --- | --- | --- | --- | --- | --- | --- | --- | --- | --- | --- | --- | --- |
| **OPG rs2073618** |  |  |  |  | **CC vs CG vs GG** |  | **CC vs (CG/GG)** |  | **(CC/CG)**  **vs GG** |  | **CG vs (CC/GG)** |  |  |  |
|  | **CC** | 29 | 78 (33.3) | 80 (46.2) | 1.00 | **0.012** | **0.57**  **(0.37-0.88)** | **0.011** | 1.08  (0.64-1.81) | 0.77 | **0.53**  **(0.34-0.83)** | **0.0051** | 0.81  (0.61-1.07) | 0.14 |
|  | **CG** | 53 | 107 (45.7) | 53 (30.6) | **0.48**  **(0.29-0.79)** |  |  |  |  |  |  |  |  |  |
|  | **GG** | 18 | 49 (20.9) | 40 (23.1) | 0.75  (0.43-1.33) |  |  |  |  |  |  |  |  |  |
| **MTHFR rs1801133**  **(C677T)** |  |  |  |  | **CC vs CT vsTT** |  | **CC VS (CT/TT)** |  | **(CC/CT)**  **vs TT** |  | **CT vs (CC/TT)** |  |  |  |
|  | **CC** | 46.9 | 141 (34.6) | 67 (35.5) | 1.00 | 0.73 | 0.91  (0.63-1.31) | 0.61 | 0.83  (0.51-1.56) | 0.46 | 1.01  (0.71-1.44) | 0.95 | 0.91  (0.70-1.17) | 0.45 |
|  | **CT** | 44.2 | 200 (49.1) | 94 (49.7) | 0.94 (0.64-1.39) |  |  |  |  |  |  |  |  |  |
|  | **TT** | 8.8 | 66 (16.2) | 28 (14.8) | 0.80 (0.47-1.38) |  |  |  |  |  |  |  |  |  |
| **MTHFR rs1801131**  **(A1298C)** |  |  |  |  | **AA vs AC vs CC** |  | **AA vs (AC/CC)** |  | **(AA/AC)**  **vs CC** |  | **AC vs (AA/CC)** |  |  |  |
|  | **AA** | 43.4 | 198 (48.6) | 88 (46.6) | 1.00 | 0.27 | 1.09  (0.77-1.55) | 0.63 | 1.64  (0.91-2.97) | 0.11 | 0.92  (0.65-1.32) | 0.65 | 1.16  (0.89-1.53) | 0.28 |
|  | **AC** | 45.1 | 176 (43.2) | 80 (42.3) | 1.00 (0.69-1.45) |  |  |  |  |  |  |  |  |  |
|  | **CC** | 11.5 | 33 (8.1) | 21 (11.1) | 1.64 (0.88-3.05) |  |  |  |  |  |  |  |  |  |

**Supplementary Table6.** Prevalence of the OPG rs2073618 and the MTHFR rs1801133 and rs1801131 SNP genotypes in RA anti-CCP positive and /or RF positive patients (Seropositive RA) and anti-CCP negative and RF negative patients (Seronegative RA), adjusted by gender and age. Hardy-Weinberg equilibrium for control group p-value: 0.06, 0.61 and 0.61 for each SNP, respectively. Genotypes, OR and p-value for the five genetic models (codominant, dominant, recessive, overdominant and additive) were estimated with SNPstats software (statistically significant if p < 0.05). AIC lowest value for OPG rs2073618 SNP was in the dominant model:289.5, for MTHFR rs1801133 was in the dominant model:312.3 and for the rs1801131 SNP was in the additive model:313.0. OPG: osteoprotegerin, MTHFR: methylene tetrahydrofolate reductase, OR: odds ratio, SNP: single nucleotide polymorphism, VS: versus, RA: rheumatoid arthritis, RF: rheumatoid factor, HAPMAP: haplotype map, AIC: Akaike information criterion.

| **SNPs** | **Genotype** | **HAPMAP (European) Database (%)** | **Seronegative RA**  **n (%)** | **Seropositive RA**  **n (%)** | **OR codominant**  **model**  **[95%CI]** | **p-value** | **OR dominant**  **model**  **[95%CI]** | **p-value** | **OR recessive**  **model**  **[95%CI]** | **p-value** | **OR over dominant**  **model**  **[95%CI]** | **p-value** | **OR log**  **additive**  **model**  **[95%CI]** | **p-value** |
| --- | --- | --- | --- | --- | --- | --- | --- | --- | --- | --- | --- | --- | --- | --- |
| **OPG rs2073618** |  |  | **n=68** | **n=173** | **CC vs CG vs GG** |  | **CC vs (CG/GG)** |  | **(CC/CG) vs GG** |  | **CG vs (CC/GG)** |  |  |  |
|  | **CC** | 29 | 23 (33.8) | 80 (46.2) | 1.00 | 0.18 | 0.58  (0.32-1.04) | 0.062 | 0.75  (0.39-1.42) | 0.38 | 0.71  (0.39-1.23) | 0.26 | 0.75  (0.53-1.06) | 0.11 |
|  | **CG** | 53 | 26 (38.2) | 53 (30.6) | 0.57  (0.29-0.1.11) |  |  |  |  |  |  |  |  |  |
|  | **GG** | 18 | 19 (27.9) | 40 (23.1) | 0.58  (0.32-1.04) |  |  |  |  |  |  |  |  |  |
| **MTHFR rs1801133**  **(C677T)** |  |  | n=72 | n=189 | **CC vs CT vs TT** |  | **CC vs (CT/TT)** |  | **(CC/CT) vs TT** |  | **CT vs (CC/TT)** |  |  |  |
|  | **CC** | 46.9 | 31 (43.1) | 67 (35.5) | 1.00 | 0.58 | 1.34  (0.77-2.35) | 0.3 | 1.07  (0.49-2.35) | 0.86 | 1.28  (0.49-2.45) | 0.38 | 1.19  (0.79-1.77) | 0.41 |
|  | **CT** | 44.2 | 31 (43.1) | 94 (49.7) | 1.37 (0.76-2.47) |  |  |  |  |  |  |  |  |  |
|  | **TT** | 8.8 | 10 (13.9) | 28 (14.8) | 1.27 (0.55-2.95) |  |  |  |  |  |  |  |  |  |
| **MTHFR rs1801131**  **(A1298C)** |  |  | n=72 | n=189 | **AA vs AC vs CC** |  | **AA vs (AC/CC)** |  | **(AA/AC) vs CC** |  | **AC vs (AA/CC)** |  |  |  |
|  | **AA** | 43.4 | 31 (43.1) | 88 (46.6) | 1.00 | 0.81 | 0.86  (0.50-1.49) | 0.59 | 0.80  (0.35-1.80) | 0.59 | 0.95  (0.55-1.65) | 0.85 | 0.88  (0.59-1.30) | 0.52 |
|  | **AC** | 45.1 | 31 (43.1) | 80 (42.3) | 0.89 (0.50-1.61) |  |  |  |  |  |  |  |  |  |
|  | **CC** | 11.5 | 10  (13.9) | 21 (11.1) | 0.76 (0.32-1.79) |  |  |  |  |  |  |  |  |  |
